# Screening of cognitive impairment by general internists using two simple instruments

**DOI:** 10.1590/S1980-57642012DN06010007

**Published:** 2012

**Authors:** Alessandro Ferrari Jacinto, Sonia Maria Dozzi Brucki, Claudia Sellitto Porto, Milton de Arruda Martins, Ricardo Nitrini

**Affiliations:** 1 MD, PhD, Behavioral and Cognitive Neurology Unit, Department of Neurology, University of São Paulo School of Medicine (FMUSP), São Paulo SP, Brazil.; 2 PhD, Behavioral and Cognitive Neurology Unit, Department of Neurology, FMUSP.; 3 MD, PhD, Department of Internal Medicine, FMUSP.

**Keywords:** screening, dementia, cognitive impairment, questionnaire, functional activity, verbal fluency, general internists

## Abstract

General internists (GIs) tend to overlook cognitive impairment in the elderly. Lack of time to diagnose and/or poor knowledge on how to use screening instruments may be the reasons for this shortcoming.

**Objectives:**

To verify the efficacy of simple instruments in the screening of cognitive impairment in elders.

**Methods:**

In a previous study, 248 patients aged ≥65 that had been assisted by GIs within outpatient services of a public university hospital in São Paulo, Brazil, were evaluated. The Mini-Mental State Examination and/or the Informant Questionnaire on Cognitive Decline in the Elderly (short-IQCODE) were employed to classify patients into probable cognitively impaired cases or otherwise. Other tests and questionnaires were also applied, but were not used to perform this classification. After full assessment and consensus meetings, cases were classified into dementia, cognitively impaired not demented, and without cognitive impairment. In this study, the sensitivity and specificity of the combined use of the category fluency test (CFT) and the Functional Activities Questionnaire (FAQ) was evaluated as if used as screening instruments for the whole sample.

**Results:**

The combined use of the CFT and/or FAQ showed sensitivity of 88.3% and specificity of 76.5% in the screening of cognitive impairment for the whole sample.

**Conclusions:**

Two simple and easy-to-apply instruments showed high sensitivity and reasonable specificity, and are probably useful for the screening of cognitive impairment in the elderly in outpatient services.

## Abstract (pt)

Médicos generalistas (MGs) normalmente não detectam comprometimento cognitivo em idosos. Falta de tempo para realizar a triagem de comprometimento cognitivo e pouco conhecimento sobre a forma de realizá-la podem ser as razões desta falha.

**Objetivos:**

Verificar a eficácia de instrumentos simples no rastreio de comprometimento cognitivo em idosos.

**Métodos:**

Em um estudo prévio, 248 pacientes com idade ≥65, que tinham sido assistidos por MGs em serviços ambulatoriais de um hospital universitário público em São Paulo, Brasil, foram avaliados. O Mini-Exame do Estado Mental e/ou o Questionário com Informante sobre Declínio Cognitivo em Idosos (Short-IQCODE) foram utilizados para classificar os pacientes em casos prováveis de cognição prejudicada ou não. Outros testes e questionários também foram aplicados, mas não foram usados para esta classificação. Depois de avaliação completa e reuniões de consenso, os casos foram classificados como demência, cognição alterada não demência e sem comprometimento cognitivo. Neste estudo avaliamos a sensibilidade e a especificidade do uso de dois instrumentos, o teste de fluência verbal (TFV) e o Questionário de Atividades Funcionais (QAF), se eles tivessem sido empregados como instrumentos de rastreio para toda a amostra.

**Resultados:**

O uso combinado do TFV e/ou do QAF mostrou sensibilidade de 88,3% e especificidade de 76,5% no rastreio de comprometimento cognitivo em toda a amostra.

**Conclusões:**

Dois instrumentos simples e de fácil aplicação mostraram alta sensibilidade e razoável especificidade e são provavelmente úteis para o rastreio de comprometimento cognitivo de idosos em ambulatórios.

## INTRODUCTION

General internists (GIs) follow elderly patients at different levels of health care settings and this practice has become increasingly frequent as populations become older in many countries.^1^ Cognitive impairment is very common among elders and its early diagnosis might be important considering the possibility of potentially reversible conditions^2^ and also the prospect of receiving adequate interdisciplinary assessment and treatment when dementia due to neurodegenerative disease is diagnosed. In addition, having patients and caregivers' futures well-planned regarding legal matters is an important issue in this context.^3,4^

Previous studies have shown that GIs overlook cognitive impairment in the elderly.^5,6^ Lack of time to properly perform a global assessment in which cognitive impairment screening is one of the most important issues, or even poor knowledge about dementia might be obstacles preventing adequate diagnosis of these conditions by GIs.^7,8^

It would be useful for GIs to apply brief instruments that did not demand complex materials in their working sets. The Category Fluency Test (CFT) seems to be a useful tool in this situation.^9,10^ This test entails asking the patient to cite as many items (animals or fruits are the most used) as possible in one minute, and the instrument has been previously studied in the Brazilian population.^11,12^ Our aim in this study was to verify the sensitivity and specificity of this one-minute test in the detection of cognitive impairment in elders followed by GIs. Also, the hypothesis of whether the use of this instrument in combination with a questionnaire (the Functional Activities Questionnaire)^13^ increases the sensitivity and specificity of the screening of cognitive impairment in elders followed by GIs was tested.

## METHODS

In this study, data was drawn from a previous study assessing the accuracy of GIs in the diagnosis of cognitive impairment in the elderly, whose methods have been reported elsewhere.^14^ Briefly, 248 patients aged 65 or older that had been assisted by GIs were evaluated.

In the assessment, subjective memory complaints, medical antecedents and use of medications were recorded, and the following tests and questionnaires were employed: the Mini Mental State Examination (MMSE),^15,16^ short version of the Informant Questionnaire on Cognitive Decline in the Elderly (Short-IQCODE),^17^ Brief Screening Cognitive Battery (the Category Verbal Fluency is included in this battery),^18,19^ Functional Activities Questionnaire (FAQ),^13^ Forward and Backward Digit Span and the 15-item Geriatric Depression Scale (GDS).^20^ Short-IQCODE and/or MMSE scores were used to classify patients into probable cognitively impaired cases or otherwise using cut-off scores previously suggested for the Brazilian population. For the MMSE, cut-off scores were 18 for illiterates, 22 for those with 1-4 years of education, 24 for 5-8 years, 26 for 9-11 years and 27 for those with 12 or more years of education.^16^ For the Short-IQCODE, the cut-off score used was 3.41.^21^ Probable cases underwent neuropsychological evaluation using the Dementia Rating Scale,^22,23^ laboratory tests (blood count, thyroid hormones, syphilis serology, liver function, kidney function, vitamin B12 and folic acid levels), and a brain computed tomography (CT) scan.^23^

Final diagnoses were established in a consensus meeting with the presence of two neurologists specialized in dementia (SMDB, RN) and the geriatrician who had evaluated the patients (AFJ), using all available data. The probable cases and a sample of 53 patients considered as not cognitively impaired based on the MMSE and/or Short-IQCODE scores were evaluated on the basis of clinical data, performances on neuropsychological tests and questionnaires for all subjects, as well as laboratory and CT results for probable cases. Patients were classified into cases with dementia, cognitively impaired not demented (CIND), and without cognitive impairment (WCI).^23-25^ Of the 248 elderly patients, 52 were classified as cognitively impaired (21 had a final diagnosis of dementia, 22 CIND while nine cases were considered not cognitively impaired). All 53 individuals classified as not cognitively impaired by the screening instruments had the final diagnosis of WCI in the consensus meeting. The specificity of the screening method (MMSE and/or Short-IQCODE) was 100% whereas the sensitivity was 82.7%.

The sensitivities, specificities and cut-off scores of the category fluency test (CFT) and FAQ for the diagnosis of cognitive impairment were obtained by comparing cases with the final diagnoses of CIND or dementia against the 53 WCI cases. Subsequently, the CFT and FAQ, adopting the cut-off scores defined above, were retrospectively applied to the entire sample of 248 elders to evaluate the accuracy of the association of both instruments for the screening of cognitive impairment.

The Research Ethics Committee of the Hospital das Clínicas of the University of São Paulo Medical School, Brazil approved this study.

**Statistical analysis**. Data were analyzed using SPSS (Statistical Package for Social Sciences) version 11.5 for Windows and "R: A Language and Environment for Statistical Computing". ROC curves were used to obtain sensitivities and specificities of the CFT and FAQ. Logistic regression was employed to obtain a compounded score for the FAQ and CFT used to determine sensitivity and specificity of the combined instruments based on the ROC curve. Five comparisons were made in order to attain sensitivities and specificities of the CFT, FAQ and both instruments combined. These comparisons were CIND × WCI, dementia × WCI, dementia + CIND × WCI, dementia × CIND + WCI and dementia × CIND. The accepted level of significance was set at 0.05.

## RESULTS

Demographic data of dementia, CIND and WCI groups are shown in Table 1.

**Table 1:** Demographic data of patients with dementia, cognitively impaired not demented (CIND) and individuals without cognitive impairment (WCI).

	WCI (N=202)	CIND (N=22)	Dementia (N=21)	P*	Multiple comparisons^†^
Age (median)	70	69.5	72	0.28	D=CIND=WCI
IQI	(67-74)	(67-73.3)	(67-75.5)		
Years of schooling (median)	4	4	2	0.02	D=CIND D<WCI CIND=WCI
IQI	(2-8)	(2-8.3)	(2-4)		

* Kruskall-Wallis test;

† Dunn's post hoc test; IQI: inter quartile interval; CIND: cognitively impaired not demented; WCI: without cognitive impairment; D: dementia.

The sensitivities and specificities of the CFT, FAQ and both instruments combined, are given in Tables 2, 3.

**Table 2:** Sensitivities and specificities of FAQ, CFT, and FAQ combined with CFT.

	AUC	95% CI	P	Cut-off Scores	Sensitivity (%)	Specificity (%)
**CIND × WCI**						
FAQ	0.909	0.836-0.983	<0.001	1	90.9	89.1
CFT	0.678	0.578-0.778	0.006	11	56.4	77.3
FAQ+CFT	0.926	0.882-0.969	<0.001	-2.54	86.4	80.6
**Dementia + CIND × WCI**						
FAQ	0.945	0.902-0.988	<0.001	2	88.4	90.3
CFT	0.777	0.701-0.853	<0.001	10	71.3	69.8
FAQ+CFT	0.957	0.930-0.983	<0.001	-1.43	90.7	94

AUC: area under curve; CIND: cognitively impaired not demented; WCI: without cognitive impairment; FAQ: Functional Activities Questionnaire; CFT: Category Fluency Test.

**Table 3:** Sensitivities and specificities of FAQ, CFT, and FAQ combined with CFT.

	AUC	95% CI	P	Cut-off Score	Sensitivity (%)	Specificity (%)
**Dementia × CIND**						
FAQ	0.897	0.805-0.989	<0.001	5	90.47	63.63
CFT	0.846	0.723-0.970	<0.001	9	90.91	76.19
FAQ+CFT	0.929	0.850-1.000	<0.001	-0.63	95.2	77.3
**Dementia × CIND + WCI**						
FAQ	0.977	0.960-0.994	<0.001	4	100	92.4
CFT	0.876	0.787-0.965	<0.001	9	88.8	76.2
FAQ+CFT	0.983	0.970-0.997	<0.001	-2.24	100	94.6
**Dementia × WCI**						
FAQ	0.988	0.976-1.000	<0.001	3	100	94.03
CFT	0.88	0.793-0.968	<0.001	9	88.61	76.19
FAQ+CFT	0.989	0.979-1.000	<0.001	-2.03	100	97.5

AUC: area under curve; CIND: cognitively impaired not demented; WCI: without cognitive impairment; FAQ: Functional Activities Questionnaire; CFT: Category Fluency Test.

When distinguishing CIND from WCI, the FAQ showed higher sensitivity and specificity than the CFT, even compared with the combination of both instruments.

For distinguishing all possible types of cognitive impairment (dementia and CIND), the FAQ again had greater efficacy than the CFT, although the combination of both instruments showed higher sensitivity and specificity.

Regarding dementia and CIND, the CFT showed similar sensitivity and specificity to the FAQ. The combination of both instruments increased the efficacy of distinguishing demented from CIND individuals.

For discriminating dementia from CIND and WCI individuals, the FAQ showed greater efficacy than the CFT but the combination of both showed higher sensitivity and specificity compared to performance of each instrument alone.

For distinguishing only dementia individuals from normal subjects, the FAQ showed greater efficacy than the CFT. Similarly, the combination of both tests yielded higher sensitivity and specificity compared to performance of each test used alone.

Applied as screening instruments with the cut-off scores suggested in Table 2, the FAQ and/or CFT would have classified 38 out of the 43 with cognitive impairment as cognitively impaired after consensus (sensitivity of 88.3%). The FAQ and/or CFT would have classified as cognitively impaired 48 out of the 205 patients without cognitive impairment (specificity of 76.5%) (Table 4).

**Table 4:** Functional Activities Questionnaire (FAQ) and category fluency test (CFT) as screening instruments.

FAQ above AND/OR CFT below cut-off scores	Cognitive impairment (final diagnosis after consensus)		
	**Present**	**Absent**	**Total**
Yes	38	48	86
No	5	157	162
Total	43	205	248

(Cut-off scores: FAQ=2; CFT=10); FAQ: functional activities questionnaire; VFT=CFT: category fluency test.

Applied as screening instruments with the cut-off scores suggested in Table 2, the FAQ and CFT would have classified 38 out of the 43 with cognitive impairment as cognitively impaired after consensus (sensitivity of 88.3%). The FAQ and CFT would have classified 48 out of 205 patients without cognitive impairment as cognitively impaired (specificity of 76.5%).

## DISCUSSION

In the present study, the FAQ had greater efficacy than the CFT for distinguishing CIND from WCI individuals. The same was observed for dementia, except when distinguishing dementia from CIND individuals. All sensitivities and specificities improved when FAQ and CFT were used together.

The CFT would likely be the better instrument for use in GIs' working sets since it is quick and easy to apply.^26^

Duff et al.^10^ studied the efficacy of the CFT for detecting dementia in groups previously classified for cognitive impairment and found that the CFT was useful in the setting of their study; a cut-off score of 15 had a sensitivity of 87% and specificity of 96%. In comparison to the Duff et al. study, the present study found overall similar sensitivities but slightly lower specificities when CFT was used alone. Compared to the study by Duff et at., our study on the CFT showed lower efficacy in detecting CIND cases. Also, cut-off scores of the CFT differed, probably due to lower education in Brazilian populations. Brucki et al.^11,12^ and Caramelli et al.^27^ also studied the CFT in a Brazilian populations and proposed cut-off scores according to educational levels.

The FAQ version currently used in Brazil^28,29^ is a simple instrument and informants are usually able to complete the questionnaire without help from the physician. In the GIs' outpatient services, site attendants could hand FAQ sheets to the informant before physician's consultation and any doubt could be resolved at the end of the appointment in order to save time. In Brazil, it is uncommon for elders to visit an outpatient service without an accompanying person, usually a relative. However, when an informant is not available, patients themselves may be able to complete the FAQ.^30^

In Brazil, as in several other countries worldwide, GIs are the health care professionals who routinely follow elderly patients but this group of physicians often overlooks cognitive impairment in this population. Cross-sectional studies of primary care physicians have found that a large number of cognitive impairment cases had not been detected by GIs.^26^ There may be several reasons for cognitive impairment being overlooked by GIs^6,8^ and one explanation proposed cites insufficient training on dementia issues given to GIs on their medical graduate programs.^8^ In Canada and Australia, Lorentz et al. showed that GIs felt that applying cognitive tests in their work settings (a large number of patients and short period to attend them) was not viable since these instruments are very complex and time-consuming to apply.^31^ However, this is not the case for the simple combination of tests proposed in this work.

The most important limitation of this study was the use of cut-off scores of the CFT and FAQ obtained from part of the same sample for which sensitivity and specificity of these screening instruments were investigated. Future studies are needed applying these two screening instruments combined to another sample in order to confirm whether this association holds.

In GIs' working sets, the CFT combined with the FAQ could be useful for cognitive impairment screening in the elderly. This combination showed high sensitivity although only moderate specificity. This means that most cases of cognitive impairment would be detected by these instruments, although around one-fourth of suspected cases would be included in excess and require further evaluation.

## Figures and Tables

**Figure 1:**
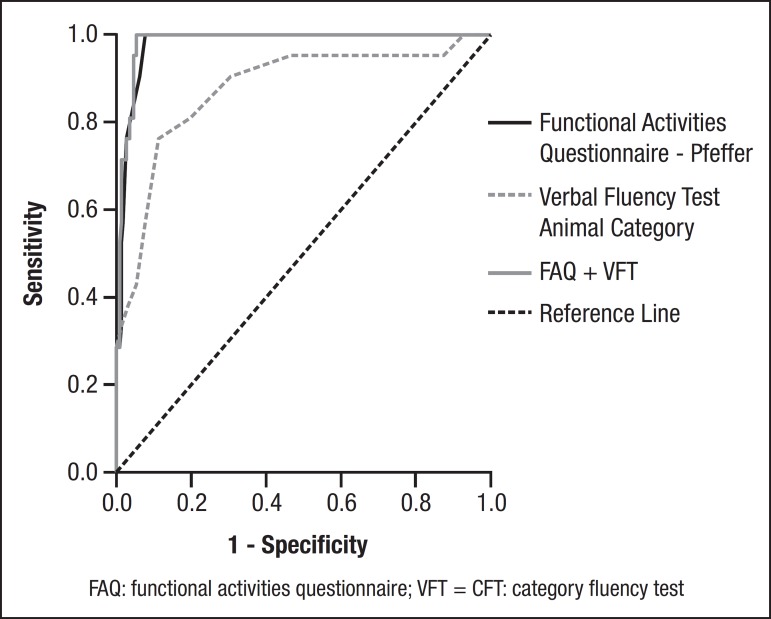
Area under the curve for dementia + CIND × WCI.
